# Impact of perinatal somatic and common mental disorder symptoms on functioning in Ethiopian women: The P-MaMiE population-based cohort study

**DOI:** 10.1016/j.jad.2011.11.028

**Published:** 2012-02

**Authors:** Vesile Senturk, Charlotte Hanlon, Girmay Medhin, Michael Dewey, Mesfin Araya, Atalay Alem, Martin Prince, Robert Stewart

**Affiliations:** a King's College London (Institute of Psychiatry), London, United Kingdom; b Department of Psychiatry, University of Ankara, Turkey; c Department of Psychiatry, School of Medicine, College of Health Sciences, Addis Ababa University, Ethiopia; d Aklilu-Lemma Institute of Pathobiology, College of Health Sciences, Addis Ababa University, Ethiopia

**Keywords:** Sub-Saharan Africa, Pregnancy, Postnatal, Somatic symptoms, Depression, Disability

## Abstract

**Background:**

Little is known of the relationship between perinatal somatic and common mental disorder (CMD) symptoms and impaired functioning in women from settings where the burden of undernutrition and infectious disease morbidity is high.

**Methods:**

A population-based sample of 1065 women from Butajira, Ethiopia, was recruited in pregnancy (86.4% of those eligible) and reassessed two months postnatal (954 with singleton, live infants). At both time-points, women were administered a modified version of the Patient Health Questionnaire-15 and the Self-Reporting Questionnaire (locally-validated) to assess somatic and CMD symptoms, respectively. Negative binomial regression was used to investigate associations of CMD and somatic symptoms with functional impairment (World Health Organisation Disability Assessment Scale, version-II), after adjusting for maternal anthropometric measures, physical ill-health and sociodemographic factors.

**Results:**

In pregnancy, somatic and CMD symptoms were independently associated with worse maternal functional impairment after adjustment for confounders (WHODAS-II score multiplied by 1.09 (95%CI 1.06, 1.13) and 1.11 (95%CI 1.08, 1.14) respectively for each additional symptom). In the postnatal period, the size of association between somatic symptoms and functional impairment was diminished, but the association with CMD symptoms was virtually unchanged (multiplier value 1.04 (95%CI 1.00, 1.09) and 1.11 (95%CI 1.07, 1.16) respectively).

**Limitations:**

Use of largely self-report measures.

**Conclusions:**

Somatic and CMD symptoms were independently associated with functional impairment in both pregnancy and the postnatal period, with CMD symptoms showing a stronger and more consistent association. This emphasises the public health relevance of both CMD and somatic symptoms in the perinatal period.

## 1 Introduction

Somatic complaints are common in the perinatal period (Brown and Lumley, 2000; Webb et al., 2008) and often assumed to be related to normal physiological changes and the physical demands associated with pregnancy, child birth and caring for a new infant. Research conducted in other populations, such as primary health care (PHC) attendees, indicates that somatic symptoms are medically unexplained in approximately one-third of cases, frequently co-occur with common mental disorders (CMD; especially depressive and anxiety disorders) (Gureje et al., 1997), and are associated with increased utilisation of health services and impaired day-to-day functioning (Kroenke et al., 2002). The few studies investigating perinatal somatic symptoms have consistently found associations with both antenatal (Kelly et al., 2001) and postnatal CMD (Ansara et al., 2005; Brown and Lumley, 2000; Josefsson et al., 2002). Somatic symptoms are more commonly reported in perinatal women compared to PHC attendees (Kelly et al., 2001). Furthermore, somatic symptoms frequently persist during the postnatal year and are associated with poorer perceived health status and quality of life (Brown and Lumley, 2000). Postnatal somatic and CMD symptoms were separately found to be associated cross-sectionally with impaired functioning in a large population-based study from the United States of America (Webb et al., 2008), but their independent effects have not been explored.

The expression of somatic complaints as a manifestation of social or psychological distress is recognised cross-culturally (Escobar and Gureje, 2007; Murphy, 1989) but in low- and middle income countries (LAMICs) its evaluation is complicated by the high burden of undiagnosed disease and undernutrition, as well as by limited access to health services. Despite this, in Indian women of reproductive age presenting to PHC, the somatic complaints of vaginal discharge and chronic fatigue were found to be more strongly associated with the presence of CMD than physical pathology, for example, the presence of infection or anaemia (Patel et al., 2005, 2006a, 2006b). For the purposes of this paper we will use the term ‘somatic symptoms’ to refer to all somatic complaints, whether or not they have been demonstrated to be medically unexplained.

Measures of perinatal CMD which include somatic items have been shown to have criterion validity in Ethiopia (Hanlon et al., 2008), Nigeria (Abiodun et al., 1993; Aderibigbe and Gureje, 1992; Uwakwe, 2003), Malawi (Stewart et al., 2008), China (Lee et al., 2001), and Mongolia (Pollock et al., 2006), as well as in high-income settings (Nott and Cutts, 1982) indicating that perinatal somatic symptoms may often be a manifestation of CMD. In Malaysia, a somatic symptom scale was strongly correlated with postnatal CMD (Grace et al., 2001). Perinatal CMD in LAMIC settings has been associated with impaired functioning (Patel et al., 2002), which has, in turn, been hypothesised to be associated with reduced help-seeking of the mother on behalf of her child (Rahman et al., 2004) and a range of poorer child health outcomes (Anoop et al., 2004; Patel et al., 2003; Rahman et al., 2004). However, little is known concerning the effect of perinatal somatic symptoms.

The relative impact of somatic and CMD symptoms on maternal functioning has not, to our knowledge, been compared in an antenatal or postnatal context in a LAMIC setting. In two cross-sectional analyses of data from a prospective community study of perinatal mental health (the *P*erinatal *Ma*ternal *M*ental Disorder *i*n *E*thiopia (P-MaMiE) study) carried out in rural Ethiopia, we aimed to (1) describe the prevalence and pattern of somatic symptoms present during pregnancy and the postnatal period and (2) to elucidate the relationship between somatic symptoms, perinatal CMD symptoms and impaired functioning at each time-point. We hypothesised that somatic symptoms at both time-points would be associated with impaired maternal functioning independent of CMD symptoms, undernutrition and reported health status.

## 2 Methods

### 2.1 Setting

Participants for the P-MaMiE study were recruited from the Demographic Surveillance Site (DSS) which is part of the Butajira Rural Health Programme (BRHP), Ethiopia (Berhane et al. 1999). Women were recruited in the third trimester of pregnancy and followed up, together with their newborn baby, shortly after birth and at two months postnatal.

### 2.2 Ethical considerations

Ethical approval was obtained from the Research Ethics Committees of the Ethiopian Science and Technology Agency (Ethiopia) and King's College London (UK). All women provided informed consent for the study. Participants were reimbursed for any health care costs for themselves and their infant from recruitment until one year postnatal, and were referred for psychiatric treatment where indicated.

### 2.3 Participants and recruitment procedures

**Fig. 1:**
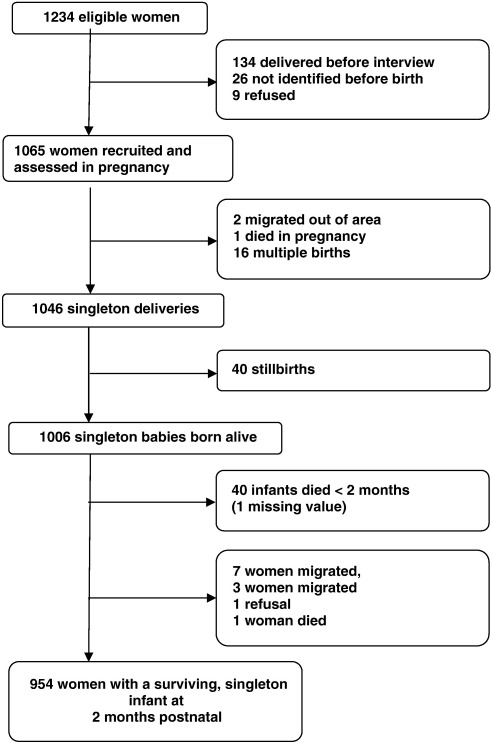
Flow chart of women with surviving singleton infants assessed at two months post-partum.

Eligible women were between the ages of 15 and 49 years, able to speak in Amharic (the official language of Ethiopia), living in the DSS and in the third trimester of pregnancy during the study recruitment period (July 2005 to March 2006). They were identified by the BRHP enumerators in the course of their three-monthly surveillance interviews and invited to participate. A flow chart of the recruitment and follow-up for the perinatal study relevant to this analysis is presented in Fig. 1. Of eligible pregnant women, 86.4% (n = 1065) chose to participate. Non-recruited women did not differ significantly from participating women in terms of age, religion, ethnicity, level of literacy, or whether they resided in urban or rural sub-districts, as reported previously (Hanlon et al., 2009a). There was minimal loss to follow-up by two months postnatal: 98.9% (n = 954) of mothers with surviving infants were assessed. For the analysis of the postnatal sample, women were excluded if they had a stillbirth, multiple birth or if the infant died before the age of two months. Measurements used in this analysis are described below. Unless stated otherwise, these were administered in identical ways at the two time-points. All measures were administered by female data collectors who had completed secondary school education and underwent rigorous training prior to commencing the study. Furthermore, data collection was carefully supervised in the field by trained supervisors, and periodic direct observation of instrument administration by the project co-investigators (CH and GM).

### 2.4 Somatic symptoms

The presence of somatic symptoms was assessed using the Patient Health Questionnaire (PHQ) (Kroenke et al., 2002) which enquires about the presence or absence and severity of fifteen symptoms commonly encountered in patients presenting to PHC settings in high-income countries. For the purposes of our analyses, the item on “menstrual cramps or other problems with your periods” was removed as it was not relevant to perinatal women. The item ‘vaginal discharge’ was used instead, given its established importance as a somatic symptom in LAMICs (Patel et al., 2006a, 2006b). Furthermore, we excluded two items that overlapped directly with depressive/anxiety symptoms, namely “feeling tired/low energy” and “problem sleeping”, that were included within our measure of CMD symptoms. As an exploratory component to our study, women were asked about four further somatic symptoms (‘burning sensation in the head’, ‘urinary problems e.g. pain’, ‘incontinence’, and (postnatally) ‘breast pain’) considered by the investigators to be commonly expressed by perinatal women in rural Ethiopia, and recognised as potential idioms of distress in previous Ethiopian studies (Youngmann et al., 1999).

### 2.5 Common mental disorder (CMD) symptoms

CMD symptoms were measured using the SRQ-20 (Beusenberg and Orley, 1994). This 20-item scale asks about depressive, anxiety and somatic symptoms present in the preceding month. This measure has been used in previous Ethiopian community-based studies (Alem et al., 1999), but was extensively pre-validated for use in perinatal women in the Butajira population (Hanlon et al., 2008). A cut-off score of ≥ 6 was shown to have convergent validity as an indicator of CMD caseness, associated with expected predictors of CMD.

### 2.6 Functional impairment

This was evaluated using the total score on the 36-item, interview-administered version of the World Health Organisation Disability Assessment Schedule II (WHODAS-II), as well as the number of working days lost in the preceding month (‘disability days’). The WHODAS-II has previously been used in population-based surveys in rural Ethiopia and shown to be acceptable and feasible (Mogga et al., 2006). The WHODAS-II consists of six domains: understanding and communication, getting around, self care, getting along with people, life activities and participation in society. Total WHODAS-II scores can range from zero to 100, with higher numbers indicating greater impairment of day-to-day functioning

### 2.7 Covariates

Age, parity and level of formal education were determined by maternal self-report. Socioeconomic status was indicated indirectly by questioning participants on the presence or absence of (i) hunger in the preceding month due to lack of money or food, (ii) subjective report of wealth relative to others, (iii) indebtedness, (iv) availability of resources to care for family for four weeks in the event of an emergency, and (v) possession of assets (ownership of land, home, business, crops, animals, bed, television, radio, cooker, jewellery or other items of value). The original list of threatening experiences (LTE) provides a measure of 12 life event categories associated with long-term threat (Brugha et al., 1985). The LTE was translated into Amharic, adapted for local conditions and the time frame restricted to the current pregnancy/time since birth. Maternal weight and height were measured using standard anthropometric techniques (WHO Expert Committee, 1995). Body mass index was calculated as weight divided by height squared (kg/m²). Women were asked about the following aspects of health during pregnancy and in the postnatal period: fever, malarial episodes and diarrhoeal episodes. Frequency of use of alcohol and khat (a popular psychoactive substance in Ethiopia with amphetamine-like effects) was categorised into ‘weekly or more’ vs. ‘less frequent use’. For obstetric complications, a composite variable was created by summing responses to the following: instrumental or operative delivery, duration of labour greater than 24 h, bleeding after delivery and fever after delivery. The resulting scale was then categorised according to number of complications: zero, one, two or more. At the postnatal examination, severity of infant illness was assessed by asking mothers whether the baby had been so ill that they thought it might die. Degree of social support was estimated by the reported frequency of contact with friends and family, as well as the reported quality of help received. Marital relationship was indicated by perceived support from husband.

### 2.8 Statistical analyses

STATA version 10 software was used for data analysis (StataCorp, 2008). Percentages and mean values, with their corresponding 95% confidence intervals (CIs), were used to summarise categorical and continuous variables respectively. Item responses on the PHQ-13 were recoded as follows: 0 = absent or present but not causing any problem, 1 = ‘bothered a little’, and 2 = ‘bothered a lot’. The total PHQ-13 scale score (main exposure) was calculated by adding the 13 individual item scores. Internal consistency of the 13-item PHQ-13 and the modified Ethiopian PHQ (16-item in pregnancy/17-item postnatally) was measured using Cronbach's alpha (Cronbach, 1951). Individual PHQ items were also dichotomised into somatic symptoms that ‘bothered a lot’ vs. less severe/absent somatic symptoms in order to calculate the prevalence of individual somatic symptoms. The association between individual somatic symptoms and high levels of CMD (SRQ-20 ≥ 6) was assessed by calculating prevalence ratios using a Poisson working model and sandwich estimates of the standard errors (Lumley et al., 2006). Spearman's rank correlation coefficient was calculated for total scores on the PHQ-13 and SRQ-20 at each time-point. Tetrachoric factor analysis using maximum likelihood, followed by varimax rotation was used to examine the constructs underlying the combined PHQ-13 and SRQ-20 scales. Scree plots of the eigenvalues at both time-points were used to determine the number of factors.

Due to the positively skewed distribution of WHODAS-II scores with prominent zero-inflation, zero-inflated negative binomial regression models (Min and Agresti, 2002) were used to investigate the association between expected covariates and WHODAS-II score as a dependent variable. Negative binomial regression is appropriate when count data are over-dispersed relative to the Poisson distribution. Zero-inflated negative binomial regression extends the model by adding an additional logistic component which models the excess of zeroes. Coefficients are on a log scale and for ease of interpretation are presented exponentiated. For example, a “multiplier” of 1.2 for the association between SRQ-20 score and WHODAS-II score indicates that for every additional SRQ symptom, the WHODAS score is increased 1.2 times (i.e. a 20% increase in the score).

The associations between exposures (PHQ-13 score/SRQ-20 score) and outcome (WHODAS-II score/‘impaired for ≥ 15 days’) variables were assessed cross-sectionally for both the pregnancy and postnatal time-points. For the outcome of total WHODAS-II score, zero-inflated negative binomial regression was used in both bivariate and multivariable analyses. Multiple logistic regression was used for the dichotomous outcome of ‘impaired for 15 days or more’. For both outcomes, the coefficients for the association between PHQ-13 or SRQ-20 score and impaired functioning are presented after (1) separate adjustments for groups of covariates, (2) full adjustment for all covariates excluding PHQ-13 or SRQ-20, and (3) full adjustment with both PHQ-13 and SRQ-20 scores included in the model.

## 3 Results

### 3.1 Sample characteristics

Characteristics of the study sample at the time of recruitment in pregnancy and at the two months postnatal time-point are shown in Table 1. Women were less likely to experience hunger due to lack of resources in the postnatal period (10.4%) compared to pregnancy (15.7%). However, they reported more ill-health postnatally: 2.3% vs. 5.8% for diarrhoeal episodes, and 13.1% vs. 25.1% for fever at the antenatal and postnatal examinations respectively. Indicators of social support were more favourable in the postnatal period.

### 3.2 Functional impairment

The distributions of WHODAS-II scores at the two examination time-points showed prominent zero-inflation. Women reported higher levels of impairment (indicated by higher WHODAS-II score) in pregnancy: median 2 (25th centile 0, 75th centile 7) compared to postnatally: median 0 (25th centile 0, 75th centile 3). Similarly, the percentage of women unable to work for 15 days or more in the preceding month was higher in pregnancy (10.6%; n = 113) compared to postnatally (7.9%; n = 75) (McNemar *χ*^2^(1) = 5.57; p = 0.018). Higher WHODAS-II scores were significantly associated with indicators of socioeconomic status, stressful life events, maternal ill-health (especially fever/malaria) and lack of help from her husband in both pregnancy and the postnatal period (See Table 1). In pregnancy, maternal age, parity and gestation at recruitment were additionally associated with higher WHODAS-II score. In the postnatal period, poorer social support from friends, obstetric complications and having a severely ill baby were also associated with higher WHODAS-II score. Drinking alcohol weekly or more frequently was associated with lower impairment in the postnatal period, although the numbers were small making the estimate unreliable.

### 3.3 Somatic symptoms

The prevalence of somatic symptoms at the two examinations are described and compared in Table 2. A similar percentage of women reported one or more PHQ somatic symptoms that bothered them ‘a lot’ in pregnancy (21.7%; n = 231) compared to the postnatal period (24.8%; n = 154). Although the percentage of women reporting backache increased postnatally (3.3% vs. 6.3%; McNemar *χ*^2^(1) 10.71; p = 0.014), there was no significant change in most somatic symptoms, apart from reductions in reported shortness of breath (1.2% vs. 0.2% McNemar *χ*^2^(1) 7.14; p = 0.013), bowel (1.3% vs. 0.2% McNemar *χ*^2^(1) 9.00; p = 0.004) and urinary problems (1.8% vs. 0.1% McNemar *χ*^2^(1) 16.20; p < 0.001), and nausea/indigestion (3.1% vs. 0.9% McNemar *χ*^2^(1) 14.40; p < 0.001). At both time-points, headache was the most commonly endorsed symptom, and dizziness, stomach pain and pains in arms, legs or joints were amongst the top five endorsed somatic symptoms at both time-points. Out of the locally-relevant somatic items, only ‘burning sensations in the head’ in pregnancy was amongst the top five most endorsed somatic items at either time-point. Overall levels of antenatal somatic symptoms (PHQ-13 score) were not associated significantly with postnatal somatic symptoms score: the multiplier of postnatal somatic symptoms for each increase in antenatal somatic symptoms was 1.03 (95%CI 0.99, 1.07).

The 13-item PHQ scale had a Cronbach's alpha of 0.75 in pregnancy and 0.68 in the postnatal period, indicating acceptable internal consistency. Addition of the locally-relevant somatic symptoms resulted in only a marginal increase in Cronbach's alpha: 0.78 for the 16-item scale in pregnancy, and 0.70 for the 17-item scale postnatally. Subsequent analyses use the original 13-item PHQ scale.

### 3.4 CMD symptoms

The prevalence of CMD (SRQ-20 ≥ 6) was greater in pregnancy (12.0%; n = 128) than in the postnatal period (4.6%; n = 44). The median SRQ-20 score was 2 (25th centile 0, 75th centile 4) in pregnancy, compared to a median score of 0 (25th centile 0, 75th centile 2) at two months postnatal. Overall levels of antenatal CMD symptoms (SRQ-20 score) were associated significantly with postnatal CMD symptom score: the multiplier of postnatal CMD symptoms for each increase in antenatal CMD symptoms was 1.12 (95%CI 1.09, 1.15).

### 3.5 Somatic and CMD symptoms

The cross-sectional associations between individual somatic symptoms and high levels of CMD (defined as SRQ-20 ≥ 6) are summarized in Table 2. All somatic symptoms from the original PHQ-13 scale were significantly associated with high CMD symptoms. Postnatally, three of the locally-relevant somatic symptoms were not significantly associated with CMD. Prospective associations between antenatal somatic symptoms (PHQ-13 score) and postnatal CMD (SRQ-20 score) were also evident: the multiplier of SRQ-20 score for each increase in somatic symptoms was 1.12 (95%CI 1.08, 1.16). Antenatal CMD was less strongly associated with postnatal somatic symptoms: the multiplier of PHQ-13 score for each increase in CMD symptoms was 1.05 (95% CI 1.02, 1.09).

The correlation between somatic symptoms (PHQ-13 score) and CMD symptoms (SRQ-20 score) was very similar in pregnancy (Spearman rank correlation coefficient 0.606; p < 0.001) and the postnatal period (correlation coefficient 0.583; p < 0.001). Despite being statistically significant, this moderate level of correlation indicated that the two scales were not measuring the same underlying construct. Furthermore, factor analysis of responses to the PHQ-13 and SRQ-20 also indicated that the scales were measuring distinct, albeit related constructs. The scree plot of eigenvalues supported a two factor solution. Items from the SRQ-20 mostly loaded onto one factor (16/20 in pregnancy and the postnatal period), and items from the PHQ-13 almost exclusively loaded onto the other factor (12/13 items in pregnancy, 11/13 in the postnatal period). Further details are available from the authors on request.

### 3.6 Cross-sectional association between somatic symptoms, CMD symptoms and impaired day-to-day functioning (Table 3)

Bivariate and multivariable analyses for the association between PHQ-13 and SRQ-20 scores and WHODAS-II score in pregnancy and the postnatal period are shown in Table 3. Both PHQ-13 score and SRQ-20 score were found to be independently associated with WHODAS-II score at both time-points, although the association between PHQ-13 and WHODAS-II was attenuated postnatally.

### 3.7 Prospective association between somatic symptoms, CMD symptoms and impaired day-to-day functioning (Table 4)

Bivariate and multivariable analyses for the prospective associations between antenatal PHQ-13 and SRQ-20 scores and postnatal WHODAS-II score are shown in Table 4. Although both antenatal SRQ-20 and PHQ-13 scores were associated with WHODAS-II score in the crude analysis, the multiplier value for antenatal PHQ-13 score became non-significant after adjusting for SRQ-20 score. The multiplier value for SRQ-20 score remained significant after adjusting for confounders and PHQ-13 separately, but became non-significant after adjusting for them simultaneously.

### 3.8 Somatic symptoms, CMD symptoms and difficulty working (Table 5)

Both PHQ-13 and SRQ-20 scores were independently associated with being unable to work for 15 or more days in the preceding month, both in pregnancy and postnatally, after adjusting for confounders.

## 4 Discussion

In this population-based study in rural Ethiopia, perinatal somatic symptoms were cross-sectionally associated with maternal CMD at both the pregnancy and postnatal time-points, but, unlike antenatal CMD symptoms, antenatal somatic symptoms were not prospectively associated with postnatal CMD. The size of the total score correlation coefficients between the PHQ-13 and SRQ-20 and factor analysis indicated that the scales were measuring distinct constructs. There was no evidence that particular somatic symptoms were more strongly associated with CMD. Both somatic symptoms and CMD symptoms were independently associated with poorer maternal functioning in cross-sectional analyses in pregnancy and in the postnatal period, after adjusting for a range of potential confounding variables. However, the prospective association between both antenatal CMD and somatic symptoms with postnatal functional impairment became non-significant in the multivariable analyses. CMD symptoms were more strongly and consistently associated with impaired functioning, both cross-sectionally and prospectively.

### 4.1 Perinatal somatic symptoms

Compared to findings from studies in high-income countries, participants in our Ethiopian sample reported far fewer somatic symptoms: in the USA, pregnant women reported a mean of 5.5 PHQ symptoms which bothered them ‘a little’ or ‘a lot’ (Kelly et al., 2001), whereas fewer than one percent of our sample reported five or more somatic symptoms at the same severity level. Given the high burden of disease and undernutrition in the Ethiopian setting, it is possible that rural Ethiopian women have a higher threshold for reporting somatic symptoms than their Western counterparts. The percentage of women reporting somatic symptoms that bothered them ‘a lot’ was almost identical in the third trimester (21.7%) and postnatal (24.8%) periods in our study, with the pattern of somatic symptoms also much the same at both time-points. This contrasts with the significant reduction in the percentage of women reporting high levels of CMD symptoms in their third trimester (12.0%) compared to the postnatal (4.6%) period. The change in CMD symptom level does not, therefore, appear to be explained by misattribution of ‘normal’ perinatal somatic symptoms to mental distress (Stewart et al., 2008).

### 4.2 Maternal functioning

The WHODAS-II score was associated with expected predictors of impaired functioning, such as maternal ill-health, pregnancy gestation, lack of social support and poverty, thus supporting the convergent validity of the scale in this population and setting. However, a high proportion of women scored zero on the WHODAS-II (“zero inflation”) which could indicate a relative lack of sensitivity to functional impairment. Other investigators working in sub-Saharan Africa have argued that impairment and disability need to be evaluated in relation to the specific socio-cultural context, with account taken of different gender roles (Bolton and Tang, 2002), and concerns have been expressed about the sensitivity of self-reported measures of functional impairment used to determine the clinical significance of mental disorder in non-Western settings (Beals et al., 2004).

Self-reported impairment of maternal functioning (reflected in the total WHODAS-II score or being unable to work for 15 days or more in the preceding month) was greater in pregnancy than in the postnatal period. This may be understandable because, by two months after childbirth, most women would be expected to have recovered from its direct effects. In addition, in Ethiopian culture, compared to pregnancy (Hanlon et al., 2010) the postnatal period is demarcated as a special time when the woman should be allowed to rest and recuperate (Hanlon et al., 2009b). As well as being exempted from physical labour and any work outside the home, the woman in the postnatal period receives better food and more help from family and neighbours. Indeed, in our study, postnatal women reported improved social support from husband, family, friends and neighbours. This may help to explain the relatively improved self-reported maternal functioning at that examination.

### 4.3 Somatic symptoms, CMD and functioning

In the cross-sectional analyses, somatic and CMD symptoms were independently associated with impaired maternal functioning in both pregnancy and the postnatal period, after adjustment for confounders. This is in keeping with findings from PHC settings in high-income countries that medically-unexplained somatic symptoms can adversely affect functioning over and above any effect they exert because of their association with CMD (Kroenke et al., 2002). Studies from other LAMIC settings indicate that somatic symptoms may require a distinctive therapeutic approach to CMD (Patel and Sumathipala, 2006; Sumathipala et al., 2008), with the potential that brief psychological interventions could lead to improved functioning as well as reductions in unnecessary help-seeking from over-stretched health facilities. Antenatal CMD symptoms were associated with postnatal functional impairment after adjusting for a range of confounders, but this association became non-significant after simultaneously adjusting for antenatal somatic symptoms. The association between antenatal somatic symptoms and postnatal functional impairment, on the other hand, became non-significant after adjusting for confounders. Taken together, these findings indicate that perinatal CMD is a stronger predictor of perinatal functional impairment, both cross-sectionally and prospectively, than perinatal somatic symptoms, although both have an independent contribution in cross-sectional analyses.

### 4.4 CMD and somatic symptoms: One construct?

Our study did find high co-morbidity between somatic symptoms and CMD, and a moderately high correlation between total somatic symptom and CMD scores, thus supporting the importance of somatisation of mental distress in this setting. In keeping with studies from high-income countries, the number of somatic symptoms rather than any specific constellation of somatic complaints appeared to be the crucial factor predicting association with CMD (Kroenke et al., 2002). However, our study also indicates that the PHQ-13 and SRQ-20 were tapping into distinct underlying constructs in this sample; specifically, the scale items loaded onto distinct factors and the scale scores were differentially associated with functional impairment.

### 4.5 Strengths and limitations

As far as we are aware, this is the first study from a LAMIC setting investigating the relative impact of perinatal somatic and CMD symptoms on maternal functioning. Population-based sampling reduced the risk of selection bias as only a minority of women (27%) attend for antenatal care in rural Ethiopia (CSA, 2006). The large sample size ensured that we were sufficiently powered to detect a true association, while enabling us to adjust for a broad range of potential confounders.

Nonetheless our study has several limitations. Our main analyses were cross-sectional thus limiting interpretation of the direction of any association, although exploratory prospective analyses are presented which support the cross-sectional findings. The use of self-report measures of physical ill-health is a further potential limitation and the lack of access to diagnostic health facilities means that there is a high burden of undetected physical ill-health within the population. We chose fever, malaria and diarrhoea as indicators of physical ill-health that were likely to be evaluated more objectively by respondents. Furthermore, maternal body mass index provides an objective non-specific indicator of general health status. The interactions between physical ill-health and CMD are, in any case, complex. Even when a physical complaint can be demonstrated, this does not rule out the presence of mental disorder, and physical disease is itself a risk factor for CMD. Having both physical and mental disorders increases the level of impairment even more, and so it remains vital to identify them both (Prince et al., 2007). Our measure of CMD, the SRQ-20, contains somatic symptoms thought to be more indicative of mental distress e.g. fatigue, sleep and appetite disturbance. These did not overlap directly with any items on the PHQ-13 but future studies may consider using measures of CMD that rely on psychological manifestations of CMD.

In conclusion, our study found that perinatal somatic and CMD symptoms often co-occur and are independently associated with impaired maternal day-to-day functioning. As well as reinforcing the importance of somatic symptoms as indicators of CMD, our study findings indicate that somatic and CMD symptoms are manifestations of distinct underlying constructs. Thus, even in the perinatal period, where somatic symptoms are often attributed to physiological changes, our study indicates that therapeutic interventions may need to be directed separately at somatic symptoms and CMD.

## Role of funding source

Funding for this study was provided by a Wellcome Trust research training fellowship in tropical clinical epidemiology for CH (Grant 071643); the Wellcome Trust had no further role in study design; in the collection, analysis and interpretation of data; in the writing of the report; and in the decision to submit the paper for publication.

## Conflict of interest

CH was supported by a Wellcome Trust research training fellowship in tropical clinical epidemiology (Grant 071643). VS was supported by a Wellcome Trust Masters Fellowship and Prize PhD Studentship. RS is funded by the NIHR Specialist Biomedical Research Centre for Mental Health at the South London and Maudsley NHS Foundation Trust and Institute of Psychiatry, King's College London. All authors declare that they have no conflicts of interest.

## Figures and Tables

**Table 1:** Characteristics of women in pregnancy and at two months postnatal, and unadjusted associations with WHO Disability Assessment Scale (WHODAS-II) score.

Characteristic	Pregnancy (n = 1065)		Postnatal 2 months (n = 954)	
	N (%) or mean (SD)	Multiplier^a^ of WHODAS-II score (95%CI)	N (%) or mean (SD)	Multiplier of WHODAS-II score (95%CI)
Maternal age (years)	26.9 (6.4)	1.02 (1.01, 1.04)	26.9 (6.3)	1.00 (0.98, 1.02)
Parity				
Nulliparous	161(15.1)	(1)	138 (14.5)	(1)
1 to 4 previous live births	596 (56.0)	1.17 (0.90, 1.54)	535 (56.1)	1.03 (0.70, 1.50)
5 or more live births	308 (28.9)	1.52 (1.14, 2.02)	281 (29.5)	1.06 (0.71, 1.58)
No formal education	846 (79.4)	0.88 (0.71, 1.09)	763 (80.0)	1.02 (0.76, 1.37)
*Socioeconomic status*				
Number of assets	4.4 (1.5)	0.92 (0.87, 0.98)	4.5 (1.5)	0.92 (0.84, 1.00)
Self-rated lower wealth	609 (57.3)	0.96 (0.80, 1.15)	539 (56.6)	1.43 (1.12, 1.82)
No emergency resources	599 (56.4)	1.09 (0.91, 1.30)	595 (62.5)	0.98 (0.76, 1.26)
Hungry from lack of money	167 (15.7)	1.41 (1.13, 1.76)	99 (10.4)	2.17 (1.61, 2.92)
Indebted	82 (7.7)	1.27 (0.96, 1.68)	77 (8.1)	1.76 (1.25, 2.50)
*Stressors*				
No. of life events				
0	632 (59.3)	(1)	672 (71.2)	(1)
1	255 (23.9)	1.23 (1.00, 1.51)	181 (19.2)	1.25 (0.97, 1.62)
≥ 2	178 (16.7)	1.75 (1.41, 2.16)	91 (9.6)	2.97 (2.17, 4.07)
*Health*				
Maternal body mass index (kg/m^2^)	21.7 (2.3) (n = 1037)	1.01 (0.97, 1.04)	20.7 (2.4) (n = 765)	1.00 (0.95, 1.05)
No. of malarial episodes				
0	886 (83.7)	(1)	824 (86.8)	(1)
1	112 (10.6)	1.41 (1.07, 1.84)	112 (11.8)	2.00 (1.46, 2.74)
2 +	60 (5.7)	1.09 (0.78, 1.53)	13 (1.4)	1.02 (0.44, 2.38)
Diarrhoea	24 (2.3)	1.58 (0.96, 2.58)	55 (5.8)	1.34 (0.88, 2.03)
Fever	139 (13.1)	1.95 (1.56, 2.44)	240 (25.1)	1.71 (1.35, 2.17)
*Substance misuse*				
Drinks alcohol weekly	54 (5.1)	0.76 (0.51, 1.11)	17 (1.8)	0.31 (0.13, 0.74)
Chews khat weekly	137 (12.9)	0.87 (0.67, 1.14)	95 (10.0)	1.25 (0.82, 1.89)
*Social support*				
Sees friends ≤ monthly	264 (24.8)	1.13 (0.92, 1.38)	144 (15.1)	1.45 (1.07, 1.97)
Not enough help at home	642 (60.3)	0.93 (0.78, 1.11)	366 (38.4)	1.00 (0.78, 1.27)
Not enough help with children	686 (66.2)	0.92 (0.76, 1.10)	426 (44.7)	0.97 (0.76, 1.23)
Not enough help from husband	124 (11.7)	1.28 (1.00, 1.64)	93 (9.9)	1.53 (1.06, 2.21)
Gestation at recruitment				
7 months	494 (46.4)	(1)		
8 months	330 (31.0)	1.33 (1.08, 1.63)	–	–
9 months	241 (22.6)	1.41 (1.13, 1.74)		
Obstetric complications				
None	–	–	330 (35.9)	(1)
One			319 (34.7)	1.09 (0.81, 1.47)
Two or more			271 (29.5)	1.36 (1.01, 1.83)
Severe infant illness	–	–	198(20.8)	1.58 (1.20, 2.08)

WHODAS-II = WHO Disability Assessment Schedule.

a A multiplier of 1.02 increases the WHODAS score 1.02 times (absolute increase of 2%) for every one year increase in the woman's age.

**Table 2:** Unadjusted associations between individual somatic symptoms (the PHQ scale + 4 somatic idioms) and high levels of common mental disorder symptoms (Self Reporting Questionnaire (SRQ-20) score ≥ 6) in pregnant and postnatal women.

Somatic item	Pregnancy				Postnatal 2 months			
	Prevalence (n, %)			Prevalence	Prevalence (n, %)			Prevalence
	Total sample n = 1065	SRQ-20 < 6 n = 937	SRQ-20 ≥ 6 n = 128	Ratio (95% CI)	Total sample n = 954	SRQ-20 < 6 n = 910	SRQ-20 ≥ 6 n = 44	Ratio (95%CI)
Stomach pain	58 (5.5)	31(3.3)	27 (21.1)	4.6 (3.3, 6.5)	46 (4.8)	40 (4.4)	6 (13.6)	3.1 (1.4, 7.0)
Back pain	35 (3.3)	17(1.8)	18 (14.1)	4.8 (3.3, 6.9)	60 (6.3)	51 (5.6)	9 (20.5)	3.8 (1.9, 7.6)
Pain in arms, legs or joints	43 (4.0)	24(2.6)	19 (14.9)	4.1 (2.8, 6.1)	47 (4.9)	39 (4.3)	8 (18.2)	4.3 (2.1, 8.7)
Vaginal discharge	5 (0.5)	1 (0.2)	4 (3.1)	6.8 (4.3, 10.9)	8 (0.8)	4 (04)	4 (9.1)	11.8 (5.6, 25.3)
Headache	120 (11.3)	66 (7.1)	54 (42.2)	5.7 (4.3, 7.7)	134 (14.0)	115 (12.6)	19 (43.2)	4.7 (2.6, 8.2)
Chest pain	22 (2.1)	9 (1.0)	13 (10.2)	5.4 (3.6, 7.9)	11 (1.2)	4 (0.4)	7 (15.9)	16.2 (9.4, 28.1)
Dizziness	52 (4.9)	32 (3.4)	20 (15.6)	3.6 (2.4, 5.3)	53 (5.6)	41 (4.5)	12 (27.3)	6.4 (3.5, 11.7)
Fainting spells	4 (0.4)	1 (0.1)	3 (2.3)	6.4 (3.5, 12)	44 (4.1)	0	44 (4.1)	–
Feeling heart pound/race	18 (1.7)	7 (0.8)	11 (8.6)	5.5 (3.6, 8.2)	12 (1.3)	9 (1.0)	3 (6.8)	5.8 (2.1, 16.0)
Shortness of breath	13 (1.2)	6 (0.6)	7 (5.5)	4.7 (2.8, 8.0)	2 (0.2)	2 (0.2)	0	–
Painful sexual intercourse	13 (1.2)	7 (0.8)	6 (4.7)	4.0 (2.2, 7.3)	8 (0.8)	5 (0.6)	3 (6.8)	8.7 (3.4, 22.3)
Bowel problems^a^	14 (1.3)	8 (0.9)	6 (4.7)	3.7 (2.0, 6.9)	2 (0.2)	0	2 (4.6)	22.7 (16.9, 30.5)
Nausea, gas or indigestion	33 (3.1)	17 (1.8)	19 (14.8)	5.0 (4.0, 7.1)	9 (0.9)	7 (0.80)	2 (4.6)	5.0 (1.4, 17.6)
Burning sensations in head^b^	36 (3.4)	16 (1.7)	17 (13.3)	4.8 (3.3, 7.0)	32 (3.4)	22 (2.4)	10 (22.7)	8.5 (4.6, 15.6)
Urinary problems^b^	19 (1.8)	8 (0.9)	11 (8.6)	5.2 (3.4, 7.9)	1 (0.1)	1 (0.1)	0	–
Incontinence^b^	1 (0.1)	0	1 (0.8)	8.4 (7.1, 9.9)	1 (0.1)	1 (0.1)	0	–
Breast pain^b^	–				23 (2.5)	21 (2.3)	2 (4.7)	1.9 (0.5, 7.6)

a Constipation, loose bowels or diarrhoea.

b Local somatic idioms, not part of original PHQ somatic scale.

**Table 3:** Bivariate and multivariable analyses of associations between (i) Patient Health Questionnaire (PHQ-13) and (ii) Self-Reporting Questionnaire (SRQ-20) with WHO Disability Assessment Schedule (WHODAS-II) scores, in pregnancy and postnatal samples.

	Pregnancy (n = 1065)		Postnatal 2 months (n = 954)	
	PHQ–WHODAS-II Multiplier value (95%CI)	SRQ-20–WHODAS-II Multiplier value (95%CI)	PHQ–WHODAS-II Multiplier value (95%CI)	SRQ-20–WHODAS-II Multiplier value (95%CI)
Crude	1.16 (1.13, 1.19)	1.15 (1.13, 1.18)	1.16 (1.12, 1.20)	1.18 (1.14, 1.22)
Separate adjustments				
Age	1.16 (1.13, 1.19)	1.15 (1.13, 1.17)	1.16 (1.12, 1.20)	1.18 (1.14, 1.22)
No formal education	1.16 (1.13, 1.19)	1.15 (1.13, 1.18)	1.16 (1.12, 1.20)	1.18 (1.14, 1.22)
Parity	1.16 (1.13, 1.19)	1.15 (1.13, 1.17)	1.16 (1.12, 1.20)	1.18 (1.14, 1.22)
Socio-economic status	1.16 (1.13, 1.20)	1.16 (1.13, 1.19)	1.13 (1.09, 1.18)	1.16 (1.12, 1.21)
Life events	1.16 (1.13, 1.19)	1.16 (1.13, 1.18)	1.12 (1.08, 1.17)	1.16 (1.12, 1.20)
Body mass index (kg/m^2^)	1.16 (1.14, 1.19) (n = 1032)	1.16 (1.13, 1.18) (n = 1032)	1.15 (1.11, 1.20) (n = 765)	1.17 (1.13, 1.21) (n = 765)
Physical health	1.16 (1.13, 1.19)	1.15 (1.13, 1.18)	1.14 (1.10, 1.18)	1.17 (1.13, 1.21)
Substance misuse	1.16 (1.14, 1.19)	1.16 (1.13, 1.18)	1.16 (1.12, 1.20)	1.18 (1.14, 1.22)
Social support	1.16 (1.13, 1.19)	1.16 (1.13, 1.18)	1.16 (1.12, 1.20)	1.18 (1.14, 1.22)
Gestation at recruitment	1.16 (1.13, 1.19)	1.15 (1.13, 1.18)	–	–
Obstetric complications	–	–	1.16 (1.12, 1.20)	1.18 (1.15, 1.22)
Infant ill-health	–	–	1.15 (1.11, 1.20)	1.18 (1.15, 1.23)

Multivariable analyses	(n = 986)		(n = 723)	
Model 1^a^	1.16 (1.13, 1.19)	–	1.08 (1.04, 1.12)	–
Model 2^a^	–	1.16 (1.13, 1.19)	–	1.11 (1.07, 1.16)
Model 3^a^	1.09 (1.06, 1.13)	1.11 (1.08, 1.14)	1.04 (1.00,1.09)	1.11 (1.07, 1.16)

a Model 1: All covariates except SRQ-20; Model 2: All confounders except PHQ; Model 3: Fully adjusted model, including all covariates, SRQ-20 and PHQ.

**Table 4:** Bivariate and multivariable analyses of prospective associations between (i) Patient Health Questionnaire (PHQ-13) and (ii) Self-Reporting Questionnaire (SRQ-20) measured in pregnancy and WHO Disability Assessment Schedule (WHODAS-II) scores measured postnatally.

	Antenatal PHQ-13 Postnatal WHODAS-II Multiplier value (95%CI)	Antenatal SRQ-20- Postnatal WHODAS-II Multiplier value (95%CI)
Crude	1.09 (1.05, 1.12)	1.08 (1.05, 1.12)
Adjusting for antenatal PHQ-13		1.06 (1.01, 1.11)
Adjusting for antenatal SRQ-20	1.03 (0.98, 1.09)	
Multivariable analyses (n = 723)		
Model 1:	1.05 (1.02, 1.08)	
All confounders except SRQ-20 Model 2		1.04 (1.01, 1.07)
All confounders except PHQ Model 3	1.04 (0.99, 1.09)	1.01 (0.96, 1.06)
Fully adjusted model, including all confounders, SRQ-20 and PHQ		

**Table 5:** Bivariate and multivariable analyses of the associations of Patient Health Questionnaire (PHQ-13) and Self-Reporting Questionnaire (SRQ-20) scores with the outcome of “percentage of women unable to work for 15 or more days in the last month”.

	Pregnancy (n = 1065)		Postnatal 2 months (n = 954)	
	PHQ—>15 days odds ratio (OR) (95%CI)	SRQ-20—>15 days OR (95%CI)	PHQ—>15 days OR (95%CI)	SRQ-20—>15 days OR (95%CI)
Crude OR	1.33 (1.25, 1.42)	1.26 (1.20, 1.33)	1.44 (1.32, 1.57)	1.38 (1.27, 1.50)
Separate adjustments				
Age	1.33 (1.25, 1.42)	1.26 (1.20, 1.33)	1.44 (1.32, 1.57)	1.38 (1.27, 1.49)
Parity	1.34 (1.25, 1.42)	1.26 (1.20, 1.33)	1.43 (1.31, 1.56)	1.39 (1.28, 1.51)
No formal education	1.33 (1.25, 1.42)	1.26 (1.20, 1.33)	1.44 (1.32, 1.57)	1.38 (1.27, 1.50)
Socioeconomic status	1.37 (1.28, 1.47)	1.31 (1.23, 1.39)	1.42 (1.30, 1.56)	1.37 (1.25, 1.50)
Life events	1.33 (1.24, 1.42)	1.27 (1.20, 1.34)	1.40 (1.28, 1.54)	1.34 (1.23, 1.46)
Body mass index (kg/m^2^)	1.34 (1.26, 1.43)	1.26 (1.20, 1.33)	1.50 (1.36, 1.66)	1.33 (1.22, 1.45)
Physical health	1.33 (1.24, 1.42)	1.26 (1.19, 1.33)	1.39 (1.27, 1.53)	1.37 (1.25, 1.50)
Substance misuse	1.34 (1.26, 1.43)	1.27 (1.20, 1.34)	1.45 (1.33,1.58)^a^	1.38 (1.27,1.50)^a^
Social support	1.35 (1.26, 1.44)	1.30 (1.22, 1.38)	1.46 (1.34, 1.60)	1.38 (1.27, 1.51)
Gestation at recruitment	1.33 (1.25, 1.42)	1.25 (1.19, 1.32)	–	–
Obstetric complications	–	–	1.44 (1.32, 1.57)	1.38 (1.27, 1.51)
Infant ill-health	–	–	1.46 (1.33, 1.59)	1.39 (1.28, 1.52)

Multivariable analyses	(n = 990)		(n = 723)	
Model 1^b^	1.39 (1.28, 1.52)	–	1.45 (1.28, 1.64)	–
Model 2^b^	–	1.30 (1.21, 1.40)	–	1.32 (1.17, 1.48)
Model 3^b^	1.27 (1.15, 1.40)	1.17 (1.08, 1.28)	1.36 (1.19, 1.55)	1.19 (1.05, 1.35)

a Khat only due to too small numbers of alcohol.

b Model 1: All confounders except SRQ-20; Model 2: All confounders except PHQ; Model 3: Fully adjusted model, including all confounders, SRQ-20 and PHQ.
